# Clarithromycin induced acute pancreatitis: A rare side effect. Case report

**DOI:** 10.1016/j.amsu.2022.103601

**Published:** 2022-04-08

**Authors:** Abdirahman Mohamed Hassan Dirie, Abdullahi Hassan Abdinur, Mohamed Abdi Osman, Mohamed Hasan Idiris

**Affiliations:** a Pulmonology Department, Mogadishu Somali Turkey Recep Tayyip Erdoğan Training and Research Hospital, Mogadishu, Somalia; b Infectious and Microbiology Department, Mogadishu Somali Turkey Recep Tayyip Erdoğan Training and Research Hospital, Mogadishu, Benadir, Somalia

## Abstract

• Rare side effect of clarithromycin.
• Drug induced acute pancreatitis.
• Macrolide induced acute pancreatitis.
• Acute pancreatitis in pneumonia treatment.

## 1 Introduction

Acute pancreatitis is defined as an acute, inflammatory, potentially life-threatening condition of the pancreas, and it is a major cause of morbidity and healthcare expenditure. There are also well-known etiologies of acute pancreatitis, among which gallstones and alcohol are the most common (40%–70% and 25%–35%, respectively). Drug-Induced Pancreatitis (DIP) is a difficult diagnosis to establish and is thus likely underreported, owing in part to its often unsuspected nature as well as the technical difficulty in causally linking a drug to acute pancreatitis cases [1]. In general, it has been estimated that medications are etiologic factors in less than 2% of acute pancreatitis [2]. Here we present a case of clarithromycin-induced acute pancreatitis while the patient was on pneumonia treatment and a dramatic decrease in enzymes after the discontinuation of clarithromycin, without changing any other drug. To our knowledge, there are only a few case reports about clarithromycin-induced acute pancreatitis in the literature worldwide, and this is the first case report in our country.

## 2 Case report

**Fig. 1:**
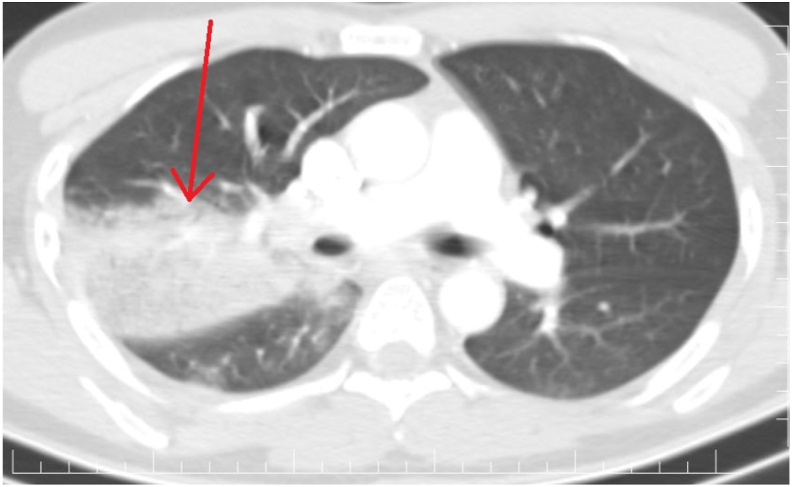
Chest CT scan with contrast parenchymal window showing consolidation in the upper lobe of the right lung (arrow).

A 31-year-old male was brought to the emergency department complaining of shortness of breath, fever, chills, pleuritic chest pain, anorexia, and cough with yellow sputum production for 2 days. He has no history of chronic diseases, smoking, drug abuse, or family history of lung diseases. On examination, the patient was in confusion and had a dry mouth. The blood pressure was 80/50 mmHg, the heart rate was 130 beats per minute, the respiratory rate was 28 cycles per minute, the temperature was 39.8 °C, and the SPO2 was 85%. On the auscultation, there were crackles in the right side of the chest. Stabilization was done in the emergency room by giving I.V fluids and a paracetamol infusion after a sample of blood was obtained. Blood tests revealed a white blood cell count of 11.4109/L with 92.4% neutrophic count, CRP: 234 mg/dL, haematocrit: 38.5%, urea 44 U/L, creatinine: 1.4 mg/dL, sodium: 145 mEq/L, and an AST of 44. CT scan with contrast was requested, which reported homogenous lobar pattern consolidation and air-bronchograms with oppasification sharply defined by the horizontal fissure of the right lung, consistence with lobar pneumonia associated with mild pleural effusion, indicating parapneumonic effusion (Fig. 1).

**Fig. 2:**
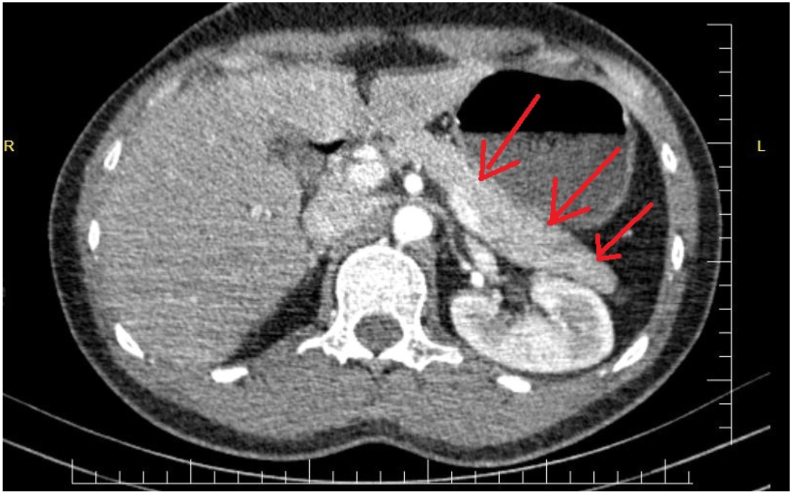
Axial abdominal CT scan with IV contrast at pancreatic level shows normal pancreas (arrows).

**Fig. 3:**
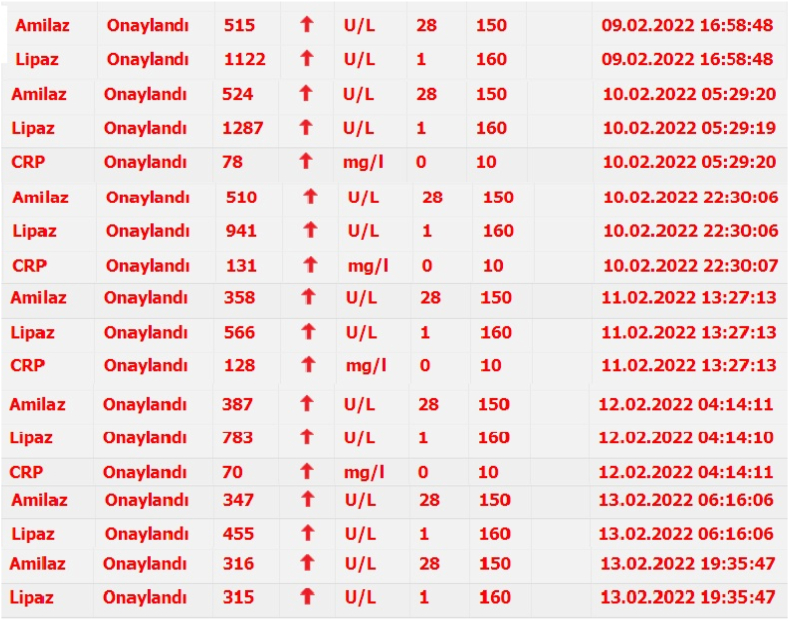
This picture is captured from computer results of pancreatic enzymes and explains the changes in enzymes from 09/02/2022 to 13/02/2022.

The patient was admitted to the ICU and IV antibiotics were started, including clarithromycin 500mg bd, ampicillin/sulbactam 1.5 g qd, *N*-acetyle cysteine 300mg tds, pantoprazole 40mg od, paracetamole infusion PRN, I.V fluids, and bronchodilators. On the third day, the patient was vitally stable, but he was complaining of nausea, vomiting, and deep abdominal pain. On the fourth day, the nausea and vomiting with abdominal pain were more severe and more frequent. So, we requested pancreatic enzymes, amylase and lipase, and the result showed lipase of 1122 U/L and amylase of 515 U/L (Fig. 3). We ruled out other causes of acute pancreatitis, including alcoholism, gallstones, and trauma. We requested an abdominal ultrasound focusing on the pancreas, and it demonstrated a normal pancreas and a normal hepato-biliary system. On the fifth day, the lipase and amylase were 1287 U/L and 524 U/L, respectively (Fig. 3), indicating that there was an active process going on despite the normal results of other investigations. We requested an abdominal CT Scan with I.V contrast, which also reported that pancreatic thickness and peri-pancreatic fat planes are normal (Fig. 2). We reviewed the literature about the side effects of all the drugs we were using for the management of the pneumonia, and we found some case reports of acute pancreatitis caused by clarithromycin. We immediately stopped the clarithromycin and continued the other drugs, and we put the patient on Non Per Oral (NPO). After 24 hours, we repeated the lipase and amylase tests, which showed a dramatic decrease in enzyme levels, 914 U/L and 510 U/L, respectively (Fig. 3). After 72 hours from stopping clarithromycin, the lipase and amylase results were 566U/L and 358U/L, respectively (Fig. 3). We reintroduced a single dose of I.V clarithromycin and checked enzymes after 6 hours of injection, but the result was impressive, showing an increase of both lipase and amylase of 783 U/L and 387 U/L, respectively. After 72 hours, lipase and amylase results were 455 U/L and 347 U/L, respectively. After 96 hours, lab results showed lipase of 383 U/L and amylase of 302 U/L. The patient otherwise improved clinically, and all lab results, including WBC, CRP, and sedimentation, returned near to the normal ranges. We discharged the patient after 7 days of hospitalization with Levofloxacin 500 mg OD. Seven days later, the patient came to our outpatient department with complete resolution of all symptoms and pancreatic enzymes within normal limits. We concluded that the offending drug that caused acute pancreatitis was clarithromycin. This work has been reported in line with the SCARE 2020 criteria [3].

## 3 Discussion

Our patient presented with symptoms and signs of severe community-acquired lobar pneumonia, which was treated by I.V antibiotics, including clarithromycin 500 mg bd. After 3 days, the patient developed signs and symptoms of acute pancreatitis with no identifiable cause other than drug-induced acute pancreatitis. The symptoms, signs, and lab results of acute pancreatitis included rapidly increasing amylase and lipase, which were concurrent with I.V clarithromycin, and a dramatic decrease of the enzymes after discontinuation of that single drug, suggesting that the clarithromycin was the cause of acute pancreatitis, mainly because there was no other drug that had been stopped or switched. Clarithromycin is an antibiotic commonly used for community-acquired pneumonia as a first-line drug with a side effect of acute pancreatitis of less than 1% as documented by the manufacturer. The mechanism by which macrolides cause pancreatitis is not clear. It has been proposed to involve direct cellular toxicity, or, that there is a direct effect on smooth muscle fibers of the intestine and that this causes spasm of the sphincter of Oddi and bile reflux [4]. In the literature, there are several case reports about clarithromycin-induced acute pancreatitis. One of the first cases of clarithromycin-induced pancreatitis was reported in 1996, in which the authors stated that symptoms resolved and amylase and lipase levels returned to normal after 3 days of discontinuing the drug [5]. In Greece, it was reported that a case of acute pancreatitis after the commencement of clarithromycin and resolution of symptoms and amylase enzyme levels after withdrawal [6](4). They documented a patient who died of suspected clarithromycin-induced acute pancreatitis, with autopsy confirming severe acute pancreatitis, albeit it remained uncertain whether clarithromycin was the cause [7]. Our case was consistent with most of the other case reports. In addition to that, we re-introduced a single dose of clarithromycin to make sure that it was the real cause of pancreatitis, and we realized that it was the offending agent. To our knowledge, this is the first case report of clarithromycin induced acute pancreatitis in Somalia.

## 4 Conclusions

Clarithromycin is one of the first-line antibiotics used for pneumonia, but one of its rare side effects is acute pancreatitis, with its common signs and symptoms. So, physicians need to be aware of this rare but fatal side effect.

## Sources of funding

There are no sponsors or any funding sources for this work.

## Author contribution

1. Abdirahman Mohamed Hassan Dirie; management and follow up of the patient, did the literature review, wrote introduction, case presentation and parts of discussion.
2. Abdullahi Hassan Abdinur; senior Pulmonologist who wrote parts of the discussion and patient Management.
3. Mohamed Abdi Osman: Made antibiotic selection and take part in the management and flow up of the patient.
4. Mohamed Hasan Idiris

## Provenance and peer review

Not commissioned, externally peer reviewed.

## Guarantor

Abdirahman Mohamed Hassan Dirie.

Abdullahi Hassan Abdinur.

## Consent

Authors have taken written consent from the patient, and it will be available on request.

## Registration of research studies

1. Name of the registry: Not applicable
2. Unique Identifying number or registration ID: Not applicable
3. Hyperlink to your specific registration (must be publicly accessible and will be checked): Not applicable

## Declaration of competing interest

Authors have no any financial or personal conflict that can influence this work.

## References

[1] Weissman S., Aziz M., Perumpail R.B., Mehta T.I., Patel R., Tabibian J.H. (2020).

[2] Ksi D. (2011).

[3] Agha R.A., Franchi T., Sohrabi C., Mathew G., Kerwan A., Thoma A. (2020). The SCARE 2020 guideline: updating consensus surgical CAse REport (SCARE) guidelines. Int. J. Surg..

[4] Avraam C., Siomos K., Armenaka M.C., Sion M.L. (2007).

[5] Care M., Park M., Mermel U., Manzella J.P., Stott G.A., Maust D. (1996).

[6] Koufakis T., Gabranis I., Ntais K., Karangelis D., Batalla S., Paschala N. (2013). Acute pancreatitis due to clarithromycin therapy: a rare adverse effect of a common drug. Eur J Intern Med [Internet].

[7] Schouwenberg B.J.J.W., Deinum J. (2003).

